# Haematological and immunological characteristics of eastern hellbenders (*Cryptobranchus alleganiensis alleganiensis*) infected and co-infected with endo- and ectoparasites

**DOI:** 10.1093/conphys/cow002

**Published:** 2016-03-21

**Authors:** William A Hopkins, Jesse A Fallon, Michelle L Beck, Brittney H Coe, Catherine M B Jachowski

**Affiliations:** Department of Fish and Wildlife Conservation, Virginia Tech, Blacksburg, VA 24061, USA

**Keywords:** Amphibian, anaemia, co-infection, leech, neutrophil-to-lymphocyte ratio, trypanosome

## Abstract

Disease is among the leading causes of the global decline in amphibian populations. In North America, parasites and pathogens are among the factors implicated in precipitous population declines of the giant hellbender salamander (*Cryptobranchus alleganiensis*), but the incidence of infections and the responses of hellbenders to infections remain poorly studied. Here, we document the prevalence of leech and trypanosome infections in a wild population of eastern hellbenders (*Cryptobranchus alleganiensis alleganiensis*) and describe haematological and immunological characteristics of hellbenders harbouring these infections. We hypothesized that hellbenders parasitized by trypanosomes would be anaemic, that individuals infected with either or both parasites would exhibit shifts in white blood cell counts and that hellbenders infected with leeches would exhibit altered plasma bactericidal capacity. We found that 24 and 68% of hellbenders in our sample population were infected with leeches and trypanosomes, respectively, and 20% were co-infected with both parasites. We found no evidence suggestive of anaemia among infected individuals. However, hellbenders infected with either or both parasites exhibited marked shifts in circulating white blood cells that were consistent with predictable responses to parasitic infection. Additionally, we found that hellbenders harbouring leeches had much higher plasma bactericidal capacity than individuals without leeches, and we offer multiple potential mechanistic explanations for this observation. We also found evidence that cellular and serological immune responses to parasites were less robust in juvenile than adult hellbenders. This finding warrants further investigation in light of the demographic characteristics, specifically the scarcity of juvenile age classes, of hellbender populations where disease is a possible contributor to declines. Finally, we describe two methodological advances that will improve future studies seeking to diagnose trypanosome infections and to test the bactericidal capacity of hellbenders and perhaps other amphibians. Our study provides fundamental insights into how hellbenders respond physiologically to endo- and ectoparasites, which could ultimately prove useful for their conservation.

## Teaser

Disease is among the leading causes of global amphibian population declines, and may contribute to declines of the giant hellbender salamander (Cryptobranchus alleganiensis). We provide some of the first information about how hellbenders physiologically respond to end- and ecto- parasites, insights that could be critical to their conservation.

## Introduction

Amphibians are among the most imperilled vertebrates in the world, and disease is one of the leading factors precipitating their population declines and species extinctions (Stuart *et al*., 2004; Hoffman *et al*., 2010). Chytridiomycosis, the disease caused by the fungal pathogen *Batrachochytrium dendrobatidis*, has received the most attention because it is probably responsible for population declines in dozens of amphibian species on multiple continents (Lips *et al*., 2006; Mendelson *et al*., 2006; Wake and Vredenburg, 2008). Many other amphibian pathogens, including *Ranavirus*, *Ichthyophonus* and *Batrachochytrium salamandrivorans*, have gained increasing attention because they can also cause significant morbidity and mortality (Mikaelian *et al*., 2000; Green *et al*., 2002; Raffel *et al*., 2006; Miller *et al*., 2011; Martel *et al*., 2014). Nonetheless, studies of sublethal effects of these and other types of infections in wild amphibians remain relatively scarce, although they could contribute significantly to our understanding of how amphibians respond to parasites and pathogens. Moreover, the response of amphibians to co-infection with multiple disease-causing organisms is seldom studied (Hoverman *et al*., 2012; Johnson and Hoverman, 2012), yet co-infection is the normal state for most wild animals (Petney and Andrews, 1998). Given that parasites can have complex interactions (e.g. inter-specific competition) within their hosts, can enhance host susceptibility to additional infections and can influence how hosts interact with their environment (Hughes *et al*., 2012; Budischak *et al*., 2015), studies are needed to understand amphibian responses to co-infections.

The giant salamanders in the Family Cryptobranchidae are a declining group of amphibians that are data deficient in terms of their basic physiology and prevalence of disease. They are critically endangered in Asia, and populations of the two subspecies in North America (Ozark hellbender *Cryptobranchus alleganiensis bishopi* and eastern hellbender *C. alleganiensis alleganiensis*) have been declining since at least the 1970s (Wheeler *et al*., 2003; Briggler *et al*., 2007b; Foster *et al*., 2009). As a result, the Ozark subspecies is now protected under the Endangered Species Act (United States Fish and Wildlife Service, 2011a), both subspecies are protected under CITES (United States Fish and Wildlife Service, 2011b), and the eastern hellbender is currently being considered for federal protection. Hellbenders are known to harbour several parasites and pathogens (Briggler *et al*., 2007a, b, 2008; Huang *et al*., 2010; Bodinof *et al*., 2011, 2012; Burgmeier *et al*., 2011; Gonynor *et al*., 2011; Regester *et al*., 2012; Souza *et al*., 2012; Tominaga *et al*., 2013), but the effects of these organisms on hellbender health and survival remain largely unknown. Recently, we discovered and described a new species of ectoparasitic leech (*Placobdella appalachiensis*) in eastern hellbenders in southwest Virginia (Hopkins *et al*., 2014). In the stream reach where this leech is currently known to occur in abundance, its prevalence of infection during late summer ranges from 21 to 48% (Hopkins *et al*., 2014; DuRant *et al*., 2015). In the same population where we discovered leeches, we also discovered a high prevalence of endoparastitic trypanosome infections (Davis and Hopkins, 2013; DuRant *et al*., 2015). Understanding how hellbenders respond to these infections and co-infections is a critical step towards ultimately determining whether disease contributes to their population declines.

Leeches and trypanosomes could have direct effects on hellbender fitness or indirectly affect their fitness by altering their physiology. For example, we demonstrated that individuals harbouring leeches are incapable of mounting a normal adrenocortical response (characterized by an increase in plasma glucocorticoids) to standardized handling restraint in the field (DuRant *et al*., 2015). Although the mechanism for this disruption is unknown, leeches release a wide variety of biologically active compounds in their saliva, including endocannabanoids, opioids and adrenocorticotrophic hormone (Salzet *et al*., 2000; Hildebrandt and Lemke, 2011), which could influence stress responsiveness and a variety of other physiological processes in hosts, including intermediary metabolism and immunity. Sanguinivorous leeches are also important vectors for other parasites and pathogens (Sawyer, 1986; Mock, 1987; Barta and Desser, 1989; Raffel *et al*., 2006) and may in fact be responsible for co-occurring hellbender infections with trypanosomes (Davis and Hopkins, 2013; DuRant *et al*., 2015). Although some trypanosomes are non-pathogenic (Linton, 1930; Grewal, 1957; Vickerman, 1969; Mansfield, 1977), others can have an array of adverse effects on their hosts, including destruction of red blood cells (RBCs) and anaemia, malaise, damage to the central nervous system, organ damage, immunosuppression and death (Meurs *et al*., 1998; Barrett *et al*., 2003; Berriman *et al*., 2005; Blum *et al*., 2006). However, the effects of trypanosomes on non-mammalian vertebrates are poorly understood (Oladiran and Belosevic, 2012). Given the sensitive status of eastern hellbenders and the fact that the federally endangered Ozark hellbender also harbours leeches (*Placobdella cryptobranchii*; Dundee and Dundee, 1965; Nickerson and Mays, 1973; Johnson and Klemm, 1977; Solís *et al*., 2007; Moser *et al*., 2008, 2013) and trypanosomes (Huang *et al*., 2010), it is crucial to describe this new host–parasite system and understand whether these parasites adversely affect hellbenders.

In the present study, we sought to determine whether infections with leeches or trypanosomes or co-infections influenced the physiology of their hellbender hosts. We hypothesized that trypanosome infection would result in symptoms of anaemia to include reduced red blood cell counts, packed cell volume (PCV) and haemoglobin (Hb) concentration of whole blood. We also predicted that if hellbenders infected with trypanosomes were anaemic, then they would have elevated numbers of young red blood cells consistent with a regenerative response. Based on our recent findings that leech infection has systemic physiological consequences for hellbenders (DuRant *et al*., 2015) and the fact that leeches produce salivary compounds that aid in evading and suppressing immune responses of their hosts (Salzet *et al*., 2000; Hildebrandt and Lemke, 2011), we hypothesized that individuals harbouring leeches would exhibit reduced plasma bactericidal capacity. Finally, we hypothesized that hellbenders infected or co-infected with parasites would display characteristic shifts in circulating white blood cells (WBCs), including an increased proportion of neutrophils and eosinophils and a decreased proportion of lymphocytes. As secondary objectives, we sought to refine our methodologies for detecting trypanosome infection and for examining the bactericidal capacity of hellbender plasma.

## Materials and methods

### Site description and sample collection

We surveyed a ∼2 km reach of stream within the Tennessee River Basin (VA, USA) where we had previously described a high prevalence of leech (*P. appalachiensis*) and trypanosome (unknown species) infections in hellbenders (Davis and Hopkins, 2013; Hopkins *et al*., 2014; DuRant *et al*., 2015). The stream drains a predominantly forested watershed (73% forest within upstream catchment of this study reach) and still harbours a relatively large population of hellbenders (∼1.8 subadult/adult hellbenders per 100 m^2^; C. M. B. Jachowski and W. A. Hopkins, unpublished observations). However, the stream is increasingly subjected to agricultural activities and suburban development, which threatens in-stream water and microhabitat quality.

We captured hellbenders over four surveys spread across 5 days (1–5 August 2013) ∼1 month prior to the onset of nesting in this stream. At this time of year, adult male and female hellbenders can be distinguished by external swelling surrounding the vent of males (Makowsky *et al*., 2010). We collected hellbenders during diurnal surveys by tactile means (colloquially termed ‘noodling’) under boulders and bedrock crevices and by turning rocks while skin-diving, which is the best method for obtaining all age classes of hellbenders (Nickerson *et al*., 2003; Nickerson and Krysko, 2003; Humphries and Pauley, 2005). All hellbenders were collected between 09.53 and 17.35 h.

Once we had captured each hellbender, we transported it quickly to the stream bank for processing. We first obtained a baseline blood sample within 3 min of initial capture (mean time for all individuals = 2:21 ± 0:09 min:s) following the methods outlined by Hopkins and DuRant (2011). We measured total length and snout–vent length (TL and SVL), weighed and sexed (based on external vent morphology of adults) each individual and subjected them to a physical examination. We counted and noted the location of leeches on hellbenders and removed several leech specimens to be preserved in 70% ethanol and archived (after Hopkins *et al*., 2014). We then injected a passive integrative transponder tag (PIT tag) into the tail musculature of each hellbender for future identification and released each individual under the rock where it was initially collected.

### Blood processing and haematology

Given that many of the haematological variables of interest are best quantified on freshly collected blood, we established a workstation along the stream bank that included areas for slide preparation, blood handling and microscopy. Equipment (e.g. Zeiss Primostar light microscope) was powered off an inverter connected to a field vehicle.

Immediately after blood collection, we made blood smears using a standard two-slide technique for trypanosome screening, RBC morphological assessment and WBC counts. For each hellbender, we prepared five slides, which were air dried and stored dry until fixing and staining in the laboratory within 1 week of collection. These slides were stained with either Azure–Xanthene stain (duplicate slides; Diff-Stain kit; IMEB, Inc.) or Wright–Giemsa stain (triplicate slides; Camco Quik Stain II) for visualizing WBCs and trypanosomes, respectively. In addition, we also prepared duplicate slides in the field using a vital stain to reveal changes in RBC morphology (Fallon *et al*., 2013). To do this, we aliquoted 20 µl of whole blood into a 0.5 ml Eppendorf vial containing 20 µl of new methylene blue stain (Ricca Chemical Co., Arlington TX, USA). The blood–stain mixture was gently mixed with the pipette tip and was incubated at ambient temperature for 20 min before being used to make slides using the standard two-slide technique.

At the same time as slides were being prepared and blood was incubating with the vital stain, we quantified total Hb (in grams per decilitre) by placing one or two drops of whole blood on a cuvette and inserting it into a Hemocue Hb Analyzer Hb201, which relies on azide–methaemoglobin reaction (Velguth *et al*., 2010). This process was repeated in duplicate for each individual, and the average reading was used in all calculations. We also aliquoted 5 μl of whole blood into a 1.5 ml Eppendorf vial containing 995 μl counting solution (Ery-TIC RBC counting kit; Bioanalytic, Germany). After repeatedly inverting the solution, we immediately filled a disposable Neubauer haemocytometer (Nexcelcom Bioscience, Lawrence, MA, USA) with 10 µl of the mixture. Given that hellbender RBCs are some of the largest by volume of any vertebrate (Jerrett and Mays, 1973), we counted all RBCs present in the nine large squares (0.9 mm^3^ total volume) at ×40 magnification on each side of the haemocytometer and averaged the two values. We calculated the total number of RBCs per microlitre of blood by multiplying by the dilution factor (200). We also filled one 75 μl haematocrit tube with whole blood from the syringe and transferred the remaining blood from the syringe into 0.5 ml microtainers containing lithium heparin (Becton Dickinson Co., Franklin Lakes, NJ, USA). The haematocrit tubes and microtainers containing whole blood were placed on ice and transported back to the laboratory.

Within 8 h of collection, haematocrit tubes were centrifuged in the laboratory at 5***g*** for 5 min. We quantified PCV for each hellbender using a standard haematocrit capillary tube reader (McCormick Scientific, St Louis, MO, USA). We calculated mean corpuscular haemoglobin concentration (MCHC; in grams per decilitre) by dividing the haemoglobin concentration of whole blood by the PCV for each individual. We also calculated mean cell volume (MCV) by dividing each individual's PCV count by its respective red blood cell count. We then removed several drops of plasma from the haematocrit tube and estimated plasma protein content (in grams per 100 ml) as total solid values using a Reichert VET 360 refractometer that compensated for temperature (Reichert, INC Depew, NY, USA). Using a Hamilton syringe, we then removed the buffy coat (visible white blood cell layer between RBCs and plasma) and prepared additional triplicate slides using the standard two-slide technique that were then air dried and stained with Wright–Giemsa. We made these buffy coat slides because trypanosomes are known to fractionate with WBCs during centrifugation (Murray *et al*., 1977), raising the possibility of enhanced detection of trypanosomes in the concentrated buffy coat layer compared with whole blood (Chappuis *et al*., 2005).

We centrifuged the blood stored in microtainers at 5***g*** for 5 min and used 20 μl of plasma for immediate determination of the bactericidal capacity of the plasma (see methods below). Remaining plasma from each individual was then aliquoted into multiple (two or three depending on plasma volume available), sterilized 0.5 ml Eppendorf tubes that were frozen and stored at −80°C. These replicated subsamples from each individual were used to determine whether the bactericidal capacity of hellbender plasma was stable after storage.

### Trypanosomes

To determine whether hellbenders were infected with trypanosomes, we examined slides stained with Wright–Giemsa with a light microscope at ×400 magnification. All slides were examined by a single observer (J. A. Fallon) who was blinded to the identity of each slide. We used three techniques to determine which technique was best for detecting trypanosome infections. First, we examined 50 random fields on standard blood smears for the presence of blood parasites (Davis and Hopkins, 2013; DuRant *et al*., 2015). Second, we re-examined the same slides but included 200 random fields of view. Finally, we examined 50 random fields of view on slides prepared from the buffy coat. In all three cases, the number of trypanosomes counted for each individual is hereafter referred to as its trypanosome infection intensity (per 50 or 200 fields of view). If no parasites were found using any of these three techniques, the animal was categorized as uninfected.

### Bactericidal capacity of plasma

We examined innate immunity in hellbenders by evaluating the bactericidal capacity of plasma (Liebl and Martin, 2009). We selected *Escherichia coli* for our immune challenges because it is a common pathogen in streams where hellbenders occur and is thus ecologically relevant. We first optimized the bactericidal assay by evaluating the ability of freshly collected plasma diluted 1:5, 1:10 and 1:20 to destroy *E. coli* at concentrations of 10^4^, 10^5^ and 10^6^ colony-forming units. The absorbance of samples was recorded after 4, 8, 12 and 24 h of incubation, and we plotted the change in bactericidal capacity over time. We found that 1:10 plasma dilution with 10^5^*E. coli* concentration and 8 h of incubation resulted in ∼50% bacteria killing.

Fresh plasma samples were run in triplicate in all assays. We diluted 3.6 μl of plasma with 32.4 μl of sterile phosphate-buffered saline (1:10 dilution) and added 12.5 μl of 10^5^ bacteria/ml *E. coli* (ATCC 8739, Epower microorganisms; Microbiologics^®^, St Cloud, MN, USA) solution to each tube and vortexed each sample. Samples were incubated at 20°C (approximating the high water temperature at this stream site in August) for 1 h, after which 250 μl of tryptic soy broth (TSB; Sigma Aldrich, St Louis, MO, USA) was added to each tube and samples were incubated for an additional 8 h at 37°C. Positive controls were prepared in triplicate by adding 12.5 μl 10^5^ bacteria/ml to 250 μl of TSB and we prepared duplicate blanks by combining 50 μl of phosphate-buffered saline with 250 μl of TSB.

Following the 8 h incubation, samples were vortexed and a Nanodrop Spectrophotometer (ND-1000; Thermo Scientific, Pittsburgh, PA, USA) was used to measure the absorbance of each sample at an optical density of 300 nm (Liebl and Martin, 2009). The absorbance of each sample and the positive controls were each averaged and used to calculate the proportion of bacteria killed as 1 − (average sample absorbance/average positive control absorbance). The Nanodrop arm was cleansed between each sample with 70% ethanol, and the entire work area was cleansed with ethanol and 10% bleach solution before and after each workday.

To examine the effects of freezing on bactericidal activity, we ran subsamples of plasma from a subset of hellbenders (*n* = 30) that were frozen at −80°C. Subsamples were re-analysed using the procedure described above after 3 and 8 weeks of freezing. Subsamples were only defrosted once, immediately before being analysed.

### Cytology

We examined slides stained with an Azure–Xanthene stain at ×400 magnification to estimate total WBC count and identified leucocytes as neutrophils, lymphocytes, eosinophils, basophils and monocytes, following Turner (1988), Hadji-Azimi *et al*. (1987), Thrall *et al*. (2004) and Campbell and Ellis (2007) . All slides were examined by a single observer (J. A. Fallon) who was blinded to the identity of each slide. At least 100 leucocytes were counted, and only fields of view with even distributions of RBCs were used. The relative number (i.e. proportion) of each cell type was calculated based on the number of cells of that type divided by the total number of leucocytes counted. The neutrophil-to-lymphocyte ratio was calculated from the proportions of these cell types. We did not estimate absolute WBC counts from blood smears because this technique has not been validated in hellbenders. We noted slight to moderate erythrocytic polychromasia in all individuals, which suggests that erythrocytes in the circulation were of varied maturity. Monocytes and basophils were in very low abundance (each <0.5% overall), and thrombocytes could not be enumerated accurately because of clumping and are therefore not discussed further.

We examined slides prepared with new methylene blue stain at ×400 magnification to characterize red blood cell morphology further, to identify vital-staining inclusion bodies and to distinguish immature RBCs (reticulocytes). In other vertebrates, vital stains such as new methylene blue can be useful for evaluating regenerative responses to anaemia by highlighting immature erythrocytes, termed reticulocytes, and for identifying oxidative injury to RBCs (Campbell and Ellis, 2007; Fallon *et al*., 2013). New methylene blue has also been used to highlight trypanosome organisms in humans (Ferreira *et al*., 2006).

### Statistical analyses

We ran all statistical analyses in PASW v18 (Quarry Bay, Hong Kong) or Microsoft Excel and recognized statistical significance at α < 0.05. Where appropriate, we tested for normality using Kolmogorov–Smirnov tests and normal probability plots and tested for homoscedasticity using Bartlett's tests. Unless otherwise noted, we used raw data in statistical analyses.

We classified all individuals as infected or uninfected with leeches and trypanosomes. Trypanosome infection status was ultimately determined using the buffy coat slides because of their superior performance over standard blood smears (see Results). We treated infection status with each parasite as a categorical variable (yes/no) rather than as a continuous variable (reflecting intensity of infection) because both statistical approaches generated similar results, trypanosome infection intensity based on slides is less quantitative than molecular diagnostic techniques, and infection intensities were not normally distributed. Individuals were classified into three age/sex classes [adult male, adult female or juvenile (unknown sex)] based on their SVL (adults >19 cm SVL; Hopkins and DuRant, 2011) and external vent morphology. Based on our *a priori* hypotheses and the interdependence of response variables, we performed separate principal components analyses (PCAs) on RBC and WBC parameters to reduce the dimensionality of the data set. Given that RBC parameters were measured on different scales, we standardized the variables to a mean of 0 and standard deviation of 1 prior to running the PCA. When multiple principal component (PC) scores were produced, we used Varimax factor rotation to simplify the factor loadings.

Prior to running statistical models, we examined the effects of SVL and age/sex class on infection status, bactericidal capacity and PC scores to determine whether these variables should be included in the final models. Given that we used SVL to refine our age classifications and these factors are interrelated, we did not use age/sex class and SVL simultaneously in the same models, but selected one based on these preliminary analyses. We determined that age/sex class described significant variance in WBCs and bacterial killing ability responses, and SVL described significant variance in RBC parameters and total plasma protein (TPP; see Results). Next, we used generalized linear models with a normal distribution and identity link function to examine the effects of leech infection (presence/absence), trypanosome infection (presence/absence), the interaction between trypanosome and leech infection, and SVL or age/sex class on RBC and WBC PC scores, bactericidal capacity and TPP. We evaluated the fit of each model using Akaike's information criteria corrected for small sample sizes (AICc). We calculated model weights and identified the 90% confidence set and used these to calculate model averaged parameter estimates and standard errors.

Finally, we used a repeated-measures ANOVA to determine whether freezing plasma at −80°C for 3 or 8 weeks affected the bactericidal capacity of hellbender plasma. We were unable to include juveniles in the repeated-measures model owing to insufficient plasma, but we did have adequate plasma to compare adult males with adult females in our model. We also assessed the repeatability of bactericidal capacity within individuals by calculating the intraclass correlation coefficient (Sokal and Rohlf, 1995).

## Results

We captured 41 hellbenders, including seven juveniles, 11 adult males and 23 adult females. Body size ranged from small juveniles (17.1 cm SVL, 85 g) to very large adults (35.7 cm SVL, 1420 g). Adult female and male hellbenders had a similar range of body sizes (*P* = 0.09). We were able to obtain a full parasite infection data set on 40 of these individuals (see below).

Hellbender erythrocytes stained with new methylene blue had no consistent reticular pattern that could be interpreted as reticulocytes, and we were therefore unable to describe regenerative responses in hellbenders. Furthermore, many erythrocytes stained with new methylene blue demonstrated folding and collapse of the cell membrane, suggesting a toxic effect of this stain on hellbender erythrocytes (e.g. Liao *et al*., 2001, 2002). Thus, future studies should attempt other vital stains, such as brilliant cresyl blue, or rely on polychromasia to characterize regenerative responses.

**Figure 1:**
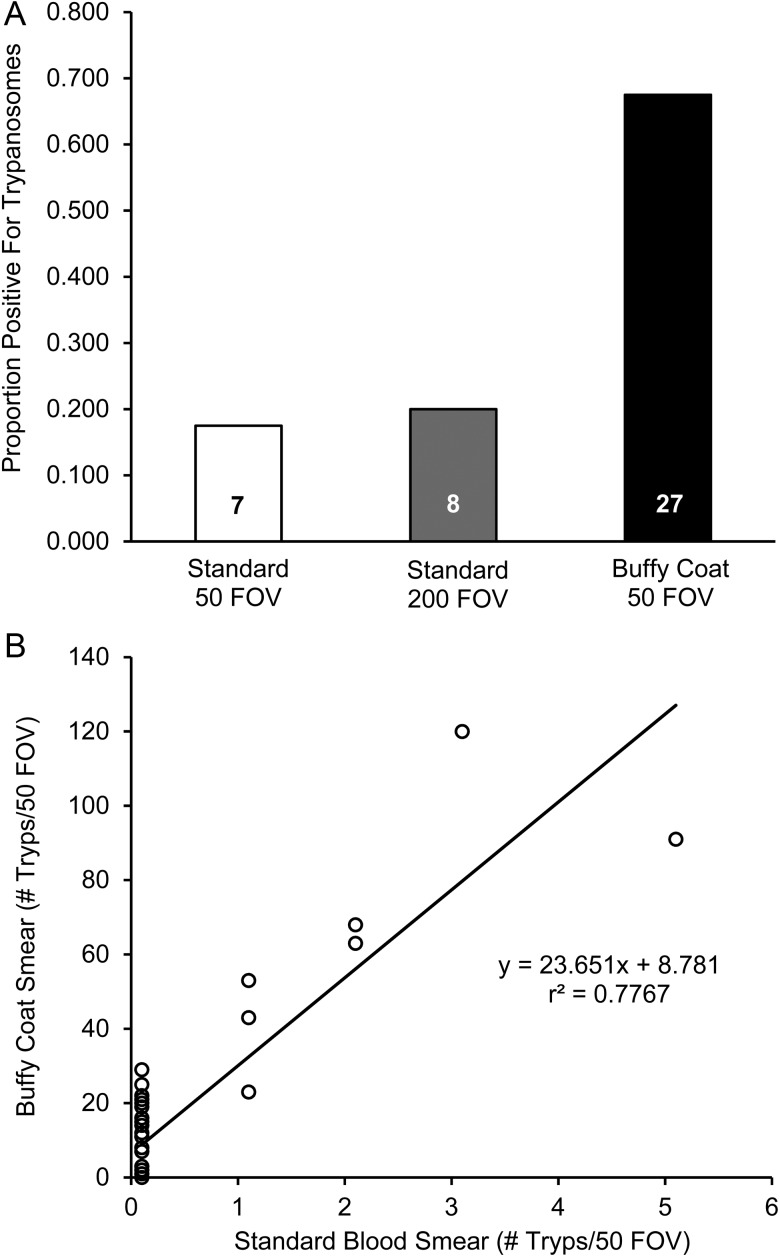
Comparison of different slide preparation techniques for detecting trypanosomes in eastern hellbenders (*Cryptobranchus alleganiensis alleganiensis*) using light microscopy. (**A**) Detection of trypanosomes is relatively poor using standard blood smears regardless of the number of fields of view (FOV) compared with smears prepared with the buffy coat. (**B**) Relationship between the number of trypanosomes (# Tryps) detected in standard smears and buffy coat smears (both from 50 FOV). Standard smears are offset slightly (+0.1) for visual purposes. Note the large range in trypanosome infection intensities that remain undiagnosed using standard smears.

**Figure 2:**
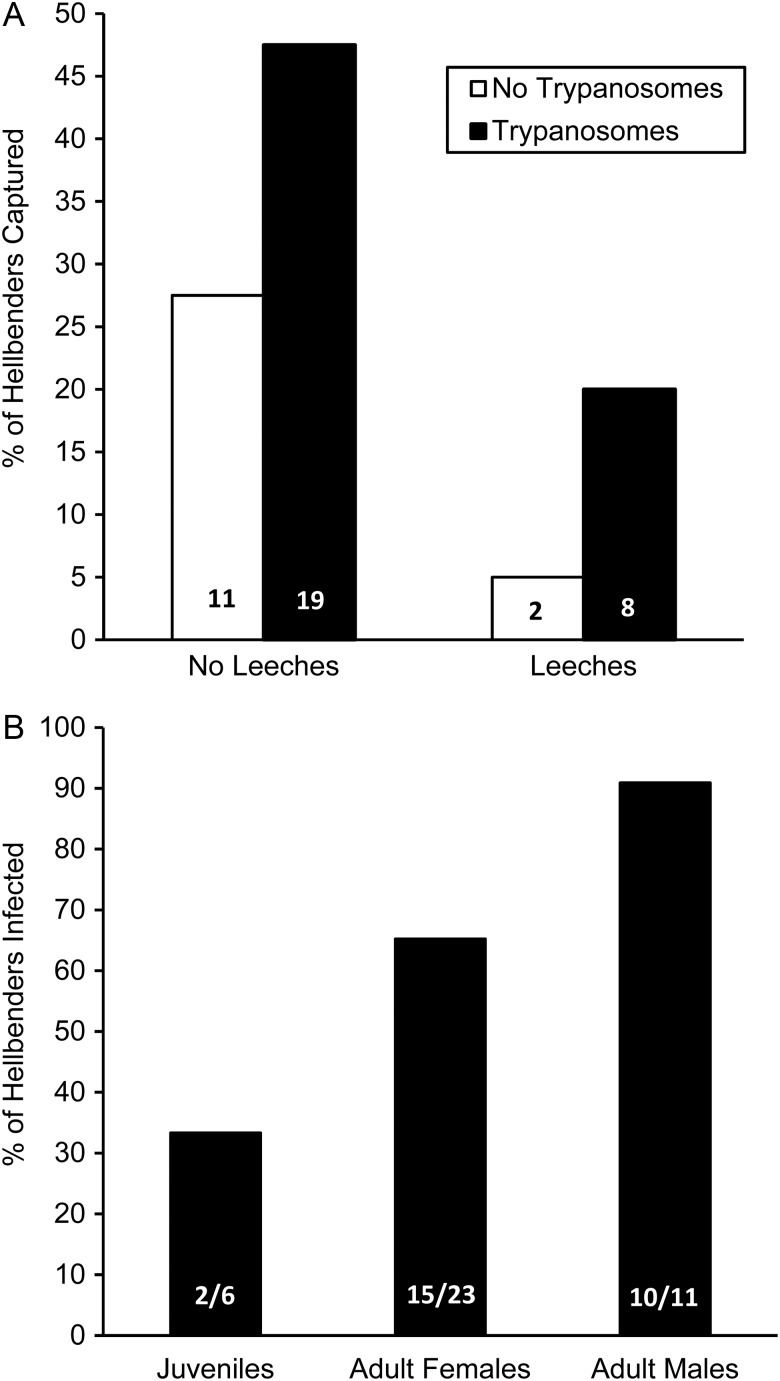
Prevalence of leech and trypanosome infections in eastern hellbenders (*C. alleganiensis alleganiensis*). Trypanosome infections were diagnosed using buffy coat smears. One individual without leeches had unreadable buffy coat slides and is not included in the figures. (**A**) Percentage of hellbenders collected that harboured leech and/or trypanosome infections. (**B**) Trypanosome infections were significantly more prevalent in adults than in juveniles.

Trypanosomes were easily recognizable when stained with new methylene blue and were consistent with our previous morphological description (Davis and Hopkins, 2013). We found that slides prepared from the buffy coat were far superior for detecting trypanosome infections compared with standard smears made from whole blood, even if the number of fields of view for standard slides was increased 4-fold (Fig. 1A). Buffy coat slides for one juvenile individual were not readable owing to poor cell dispersion, so this individual was excluded from all models involving trypanosome infection. All individuals that tested positive using the standard technique also tested positive using the buffy coat slides. However, 20 of 40 individuals were misidentified as trypanosome negative using standard blood smears, and this was true across a wide range of trypanosome infection intensities (Fig. 1B). Resultant differences in prevalence were 17.5% using the standard technique vs. 67.5% using the improved buffy coat technique. Importantly, infections were significantly more prominent in adults than in juveniles (likelihood ratio test = 6.39, *P* = 0.04; Fig. 2). However, body size alone was not a good predictor of whether individuals were infected with trypanosomes (binary logistic regression, *P* = 0.88).

Leech infections were less prevalent in our sample population than trypanosome infections. Overall, the prevalence of leech infection was 24% (10 of 41 individuals), and the median intensity of infection among individuals harbouring leeches was 1.5 (range = 1–6 leeches). We detected no difference among age/sex classes in the prevalence of leech infection even though no juveniles harboured leeches [likelihood ratio test = 4.40, *P* = 0.11; juveniles = 0% (0 of 7), adult females = 30% (7 of 23), and adult males = 27% (3 of 11)]. Likewise, SVL did not influence the probability of being infected with leeches (binary logistic regression, *P* = 0.45) or the intensity of leech infection (linear regression, *P* = 0.63).

Average RBC parameters are reported in Table 1. For these RBC parameters, our PCA produced two PC scores that together explained 80% of the variance (Supplemental Table 1). Red blood cell PC1 received high positive factor loadings for Hb, PCV (%) and RBCs and explained 48.8% of the variance, whereas RBCPC2 received a high positive factor loading for MCV and a negative loading for MCHC and explained 31.2% of the variance. Our PCA for WBC parameters produced a single PC score that received high positive loadings for the percentage of neutrophils, percentage of eosinophils and neutrophil-to-lymphocyte ratio, a negative loading for the percentage of lymphocytes and explained 74.6% of the variance (Supplemental Table 1).

**Table 1:** Haematological characteristics of eastern hellbenders (*Cryptobranchus alleganiensis alleganiensis*) from a population in southwest Virginia, USA

	Infection status					
	Neither	Trypanosomes		Leeches		Co-infected
Parameter	*n* = 11	No (*n* = 13)	Yes (*n* = 27)	No (*n* = 30)	Yes (*n* = 10)	*n* = 8
PCV (%)	29.49 ± 1.92	30.30 ± 1.53	32.12 ± 1.06	30.89 ± 0.99	32.55 ± 1.75	32.52 ± 2.25
RBC count (/ml)	68 244 ± 5019	69 421 ± 4586	64 374 ± 3182	64 860 ± 3014	66 945 ± 5326	66 068 ± 5889
Hb (g/100 ml)	7.99 ± 0.45	7.73 ± 0.41	8.43 ± 0.29	8.22 ± 0.28	7.91 ± 0.49	8.41 ± 0.53
MCV (fL)	4441 ± 266	4489 ± 249	5124 ± 173	4929 ± 170	4947 ± 301	4973 ± 312
MCHC (g/dl)	27.31 ± 0.88	25.91 ± 0.91	26.44 ± 0.63	26.78 ± 0.56	24.74 ± 0.99	26.22 ± 1.03
TPP (g/100 ml)	2.46 ± 0.11	2.69 ± 0.17	2.75 ± 0.12	2.66 ± 0.11	2.85 ± 0.19	2.63 ± 0.13
Percentage of neutrophils	25.36 ± 1.68	25.31 ± 1.52	30.67 ± 1.06	28.23 ± 1.07	30.60 ± 1.89	32.00 ± 1.98
Percentage of lymphocytes	62.09 ± 2.38	60.54 ± 2.36	50.07 ± 1.64	55.68 ± 1.64	47.30 ± 2.88	46.13 ± 2.79
Percentage of eosinophils	12.36 ± 2.19	13.92 ± 1.87	18.67 ± 1.30	15.74 ± 1.18	21.30 ± 2.08	21.00 ± 2.57
N:L Ratio	0.42 ± 0.06	0.44 ± 0.05	0.64 ± 0.04	0.54 ± 0.04	0.67 ± 0.06	0.71 ± 0.06
BKA (%; fresh)	35.30 ± 6.81	40.53 ± 6.43	48.54 ± 4.46	40.08 ± 3.82	62.19 ± 6.73	60.42 ± 7.99

Values are reported based on the infection status of individuals; note that the same individuals are recategorized for trypanosome and leech infection status. Least-squares means (±1 SEM) corrected for body size (snout–vent length) are presented for PCV, RBC, Hb, MCV, MCHC and TPP. Arithmetic means (±1 SEM) are presented for the remaining variables because body size did not influence these parameters. Abbreviations: BKA, bacterial killing ability; Hb, blood haemoglobin concentration; MCV, mean corpuscular volume; MCHC, mean corpuscular haemoglobin concentration; N:L ratio, neutrophil-to-lymphocyte ratio; PCV, packed cell volume; RBC, red blood cell; and TPP, total plasma protein.

**Figure 3:**
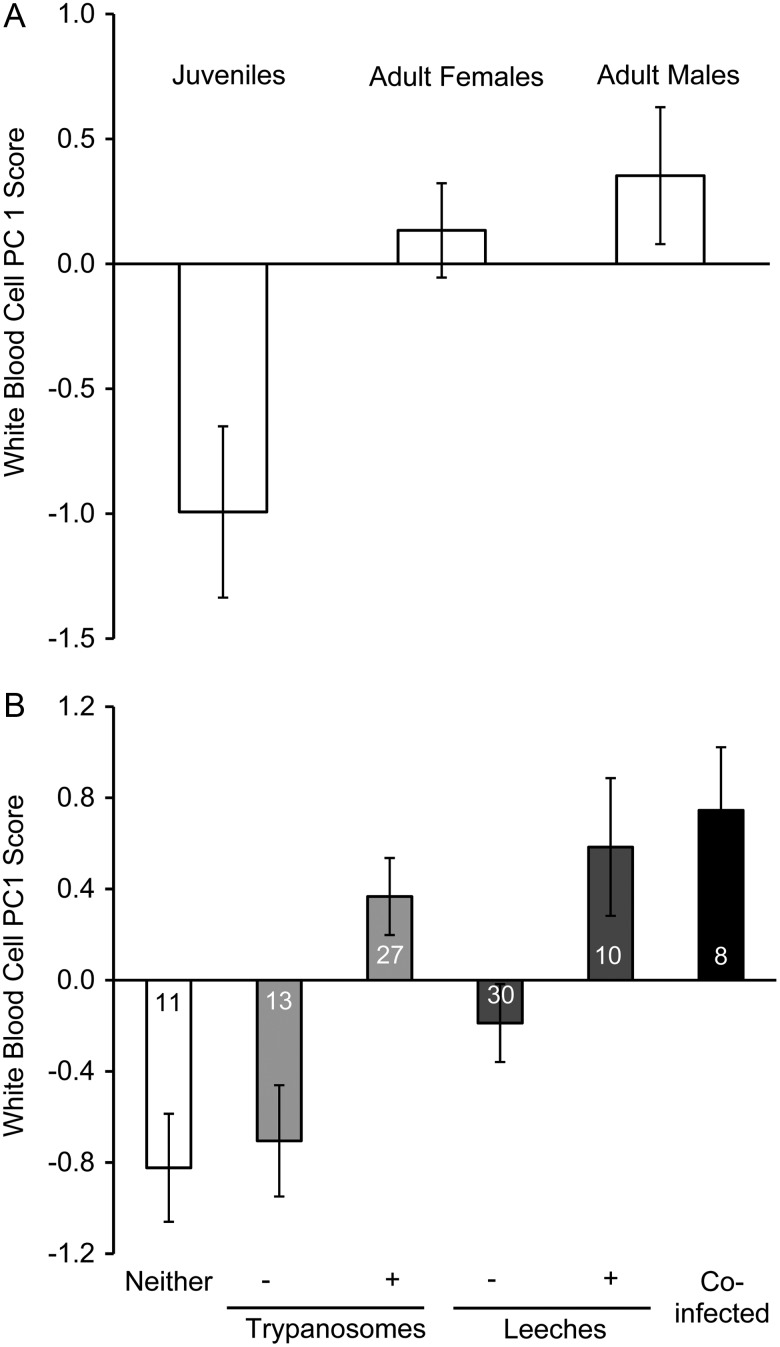
Influence of age/sex class and infection status on blood parameters related to immunity of eastern hellbenders (*C. alleganiensis alleganiensis*). (**A**) Mean (±1 SEM) white blood cell principal component 1 (PC1) scores, which included high positive factor loadings for the percentage of neutrophils, percentage of eosinophils and N:L ratio as well as a high negative loading for the percentage of lymphocytes. (**B**) Mean white blood cell PC1 scores based on the infection status of individuals. Note that the same individuals were reclassified according to their trypanosome and leech infection status.

**Figure 4:**
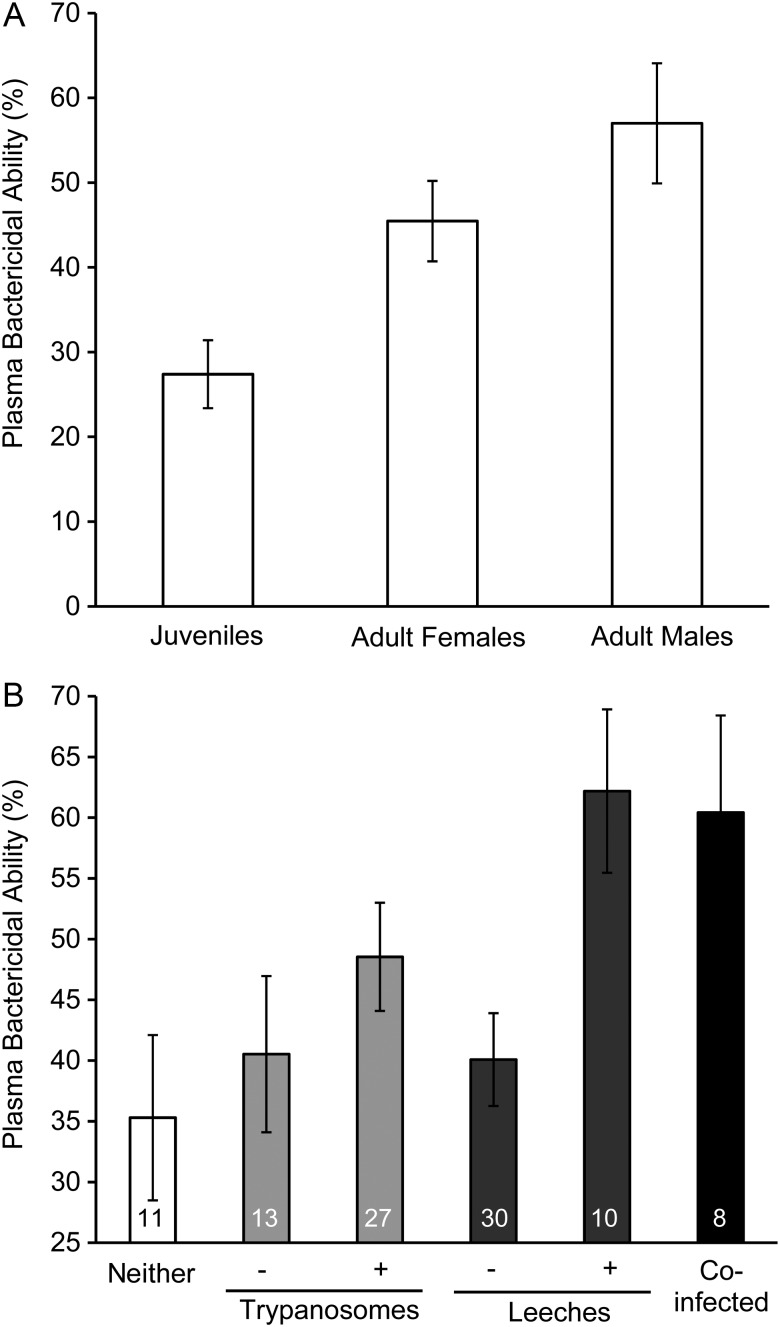
Influence of age/sex class and infection status on the bactericidal capacity of freshly collected plasma (day 0, unfrozen) from eastern hellbenders (*C. alleganiensis alleganiensis*). (**A**) Mean (±1 SEM) bactericidal capacity expressed as the percentage of killing of the three age/sex classes. (**B**) Mean (±1 SEM) bactericidal capacity based on the infection status of individuals. Note that the same individuals were reclassified according to their trypanosome and leech infection status.

In preliminary analyses, we found that RBCPC1 showed a significantly positive relationship to SVL (*r^2^* = 0.139, d.f. = 40, *P* = 0.006; SupplementalFig. 1A), as did TPP (*r^2^* = 0.422, d.f. = 40, *P* < 0.001; Supplemental Fig. 1B), whereas all other physiological response variables were unrelated to SVL (all *P* ≥ 0.19). We also found that age/sex classes differed significantly in WBCPC1 (*F*_2,38_ = 5.268, *P* = 0.01; Fig. 3A) and bactericidal capacity (*F*_2,38_ = 4.048, *P* = 0.02; Fig. 4A), and in both cases, juveniles had lower responses than adult males or females. In contrast, TPP and RBCPC1 were not influenced by age/sex even after we controlled for SVL (ANCOVAs: *P* = 0.08 and 0.12, respectively). Although RBCPC2 was not significantly related to SVL or age/sex class, we included SVL in these models for consistency because SVL affected RBCPC1.

For RBCPC1, the best model received a moderate model weight of 0.540 and included SVL (Table 2). A total of four models made up the 90% confidence set, and all four included SVL, whereas trypanosome and leech infection status occurred in the others. Model averaged parameter estimates showed that SVL had a nearly three times stronger relationship with RBCPC1 than leech or trypanosome infection status (Table 3). Larger individuals had greater RBCPC1 scores, hence greater Hb, PCV and RBC counts. No models received strong support for RBCPC2 (model weights <0.152), and the intercept-only model was the third highest ranked model (Table 2). The 90% confidence set included eight of the 10 models we ran, suggesting that none of the models explained RBCPC2 scores particularly well. Although leech and trypanosome infection status occurred in our final averaged model (Table 3), model rankings and the precision of parameter estimates provided little evidence that RBCPC2 was affected by infection status or body size of hellbenders in our sample population.

**Table 2:** Results from generalized linear model selection examining the effects of infection status, body size and age/sex class (class) on blood and immune parameters in eastern hellbenders

Response	Model	AICc	ΔAICc	Model weight
RBCPC1	β + SVL	109.72	0	0.540
	β + trypanosome + SVL	112.09	2.37	0.165
	β + leech + SVL	112.11	2.39	0.163
	β + trypanosome + leech + SVL	114.65	4.93	0.046
	β	114.90	5.18	0.041
	β + leech	116.96	7.24	0.014
	β + trypanosome	117.11	7.39	0.013
	β + leech + trypanosome + trypanosome × leech + SVL	117.42	7.70	0.012
	β + trypanosome + leech	119.36	9.63	0.004
	β + leech + trypanosome + trypanosome × leech	121.93	12.21	0.001
RBCPC2	β + SVL	117.65	0	0.152
	β + leech + trypanosome + trypanosome × leech	117.68	0.03	0.150
	β	117.79	0.14	0.142
	β + trypanosome + SVL	118.40	0.75	0.105
	β + trypanosome	118.41	0.75	0.104
	β + leech	118.73	1.07	0.089
	β + leech + trypanosome + trypanosome × leech + SVL	118.74	1.08	0.088
	β + leech + SVL	119.0	1.35	0.078
	β + trypanosome + leech	119.9	2.20	0.051
	β + trypanosome + leech + SVL	120.2	2.58	0.042
TPP	β + leech + trypanosome + trypanosome × leech + SVL	74.55	0	0.612
	β + SVL	76.70	2.15	0.209
	β + leech + SVL	78.44	3.89	0.088
	β + trypanosome + SVL	79.10	4.54	0.063
	β + trypanosome + leech + SVL	81.04	6.48	0.024
	β + leech + trypanosome + trypanosome × leech	85.95	11.39	0.002
	β	88.56	14.01	0.001
	β + leech	89.09	14.53	0.0004
	β + trypanosome	90.80	16.25	0.0002
	β + trypanosome + leech	92.20	17.65	0.0001
WBCPC1	β + trypanosome + leech	106.66	0	0.306
	β + trypanosome + class	107.54	0.88	0.197
	β + trypanosome	107.81	1.15	0.173
	β + trypanosome + leech + class	107.91	1.25	0.164
	β + leech + trypanosome + trypanosome × leech	109.18	2.52	0.087
	β + leech + trypanosome + trypanosome × leech + class	110.87	4.21	0.037
	β + leech + class	112.61	5.95	0.016
	β + class	112.77	6.11	0.014
	β + leech	115.04	8.38	0.005
	β	117.28	10.62	0.002
BKA	β + leech + class	362.65	0	0.369
	β + leech	363.39	0.73	0.256
	β + trypanosome + leech	365.36	2.70	0.095
	β + trypanosome + leech + class	365.40	2.75	0.093
	β + class	366.14	3.49	0.065
	β + leech + trypanosome + trypanosome × leech	367.09	4.44	0.040
	β + leech + trypanosome + trypanosome × leech + class	367.55	4.90	0.032
	β	368.38	5.73	0.021
	β + trypanosome + class	368.76	6.10	0.017
	β + trypanosome	369.63	6.98	0.011

Abbreviations: BKA, bacterial killing ability; RBCPC1, red blood cell principal component 1; RBCPC2, red blood cell principal component 2; SVL, snout–vent length; TPP, total plasma protein; WBCPC1, white blood cell principal component 1; AICc, Akaike's information criteria corrected for small sample sizes; and ΔAICc, the difference between a model's mean AICc value and the mean AICc value of the best fit model.

**Table 3:** Model averaged parameter estimates and standard errors from the 90% confidence set for hellbender blood and immune parameters

Response	Parameter	Parameter estimate	Standard error
RBCPC1	Leech	0.021	0.075
	Trypanosome	0.022	0.070
	SVL	0.060	0.021
RBCPC2	Leech	0.515	0.617
	Trypanosome	0.281	0.218
	SVL	0.016	0.011
	Trypanosome × leech	−0.459	0.647
TPP	Leech	0.898	0.451
	Trypanosome	0.204	0.138
	SVL	0.053	0.013
	Trypanosome × leech	−0.984	0.458
WBCPC1	Leech	0.348	0.249
	Trypanosome	0.952	0.297
	Age/sex class	−0.166	0.170
	Trypanosome × leech	−0.021	0.071
BKA	Leech	−9.395	3.898
	Trypanosome	−0.190	1.572
	Age/sex class	−3.564	5.296
	Trypanosome × leech	−0.187	0.926

Abbreviations: BKA, bacterial killing ability; RBCPC1, red blood cell principal component 1; RBCPC2, red blood cell principal component 2; SVL, snout–vent length; TPP, total plasma protein; and WBCPC1, white blood cell principal component 1.

For TPP, the highest ranked model received a moderate model weight of 0.612 and included all of the terms (Table 2). The 90% confidence set consisted of three models, and leech infection status appeared in two of the top three, whereas SVL occurred in all three. However, in the top model, leech and leech × trypanosome infection status had the greatest parameter weights, which led them to receive the highest model averaged parameter estimates (Table 3). Individuals with leech infections had 7 and 15% higher TPP concentrations than individuals without leech infections and completely uninfected individuals, respectively (Table 1).

The 90% confidence set for WBCPC1 consisted of five models; all five models included trypanosome infection status, three of the five included leech infection status, and two of the top models included age/sex class (Table 2). Model averaged parameter estimates suggested that trypanosome infection status had the strongest relationship with WBCPC1 scores followed by leech infection status (Table 3). Trypanosome and leech infection both had a strong association with WBCPC1 scores (Fig. 3B). Specifically, infected individuals had a higher percentage of neutrophils and eosinophils, a lower percentage of lymphocytes and a higher N:L ratio than individuals not infected with trypanosomes (Table 1).

For bactericidal capacity, the top model included leech and age/sex class, and the second model included only leech (Table 2). The 90% confidence set consisted of six models; leech infection status appeared in five of the six models, and age/sex class appeared in three of the six models. Model averaged parameter estimates indicated that leech infection status had the strongest association with bactericidal capacity followed by age/sex class (Table 3). Individuals infected with leeches had 55% higher bactericidal capacity than individuals uninfected by leeches (62% vs. 40% killing capacity, respectively; Table 1 and Fig. 4B).

**Figure 5:**
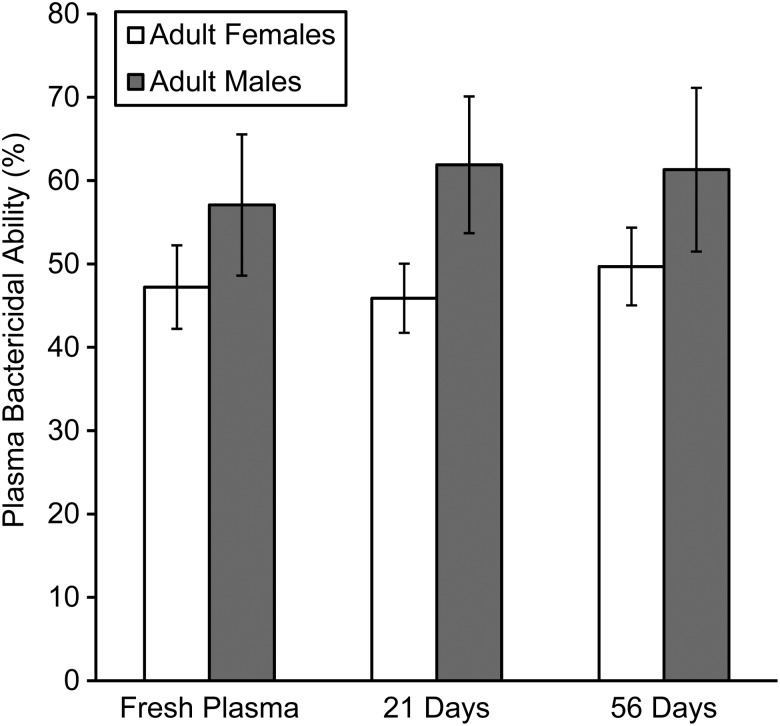
Stability of bactericidal capacity of plasma after storage at −80°C. Plasma was collected from eastern hellbenders (*C. alleganiensis alleganiensis*) and bactericidal capacity determined in fresh plasma (day 0) and again after 21 and 56 days in the freezer. The figure displays the mean (±1 SEM) of *n* = 21 adult females and nine adult males at all three time points.

We found that hellbender plasma frozen at −80°C for 3 and 8 weeks maintained remarkably stable bactericidal capacity compared with fresh plasma (time: *F*_2,27_ = 0.49, *P* = 0.615; Fig. 5). In addition, we found that male and female hellbenders had similar bacterial killing capacity, and plasma from both sexes behaved in a similar manner over time in the freezer (sex: *F*_1,28_ = 2.22, *P* = 0.148; sex × time: *F*_2,27_ = 0.66, *P* = 0.523; Fig. 5). We also found that bactericidal capacity within individuals was highly repeatable over time [intraclass correlation coefficient (95% confidence interval) = 0.785 (0.652–0.881)].

## Discussion

Our study provides fundamental insights into the incidence of two potentially detrimental parasites and the correlated physiological responses of their imperilled amphibian hosts. We found that 73% of hellbenders in our sample population were infected with leeches and/or trypanosomes. Individuals infected with parasites exhibited shifts in leucocyte profiles and plasma protein concentrations that were consistent with responses to parasitic infection, and contrary to our initial predictions, they also displayed increased plasma bactericidal ability. However, we found no evidence that parasites influenced haematological parameters indicative of anaemia. Taken together, our findings suggest that leeches and trypanosomes provoke physiological responses in hellbenders, but future work is still needed to determine whether these parasites are detrimental to the fitness of their hosts.

The prevalence (24%) and intensity (one to six leeches per individual) of leech infection documented here was within the range of what we observed in previous years at this same study site (21–48% prevalence, range of one to >250 leeches; Hopkins *et al*., 2014; DuRant *et al*., 2015). The prevalence of leech infections in the present study was statistically similar among age/sex classes and across body sizes. However, no juveniles harboured leeches, a finding that is qualitatively consistent with our previous work that suggested infection intensity might increase with body size (Hopkins *et al*., 2014). As both of these data sets are relatively small (*n* = 10–22 individuals with leeches), a more expansive multiyear data set will ultimately be required to determine the relationship between body size and leech parasitism. Nevertheless, our work at this site suggests that *P. appalachiensis* is consistently common on their eastern hellbender hosts in this system, but that they occur much less frequently than the closely related leech *P. cryptobranchii* that parasitizes Ozark hellbenders (up to 96% prevalence; Nickerson and Mays, 1973; Johnson and Klemm, 1977; Solís *et al*., 2007; Moser *et al*., 2008). The reasons for such large differences in leech prevalence between Ozark and eastern hellbenders are unknown. Regardless, circumstantial evidence based on spatial co-occurrence of leeches and trypanosomes in both Ozark and eastern hellbenders suggests that leeches may serve as vectors for these blood parasites (Huang *et al*., 2010; W. A. Hopkins and C. M. B. Jachowski, unpublished observations). Ultimately, a controlled transmission experiment is needed to provide definitive evidence that leeches act as vectors for trypanosomes among their hellbender hosts.

We demonstrated that examining 50 fields of view on slides prepared from the buffy coat increased our detection 4-fold over whole blood smears (from 17.5 to 65%; Fig. 1A) over a wide range of infection intensities (1–30 trypanosomes per 50 fields of view; Fig. 1B). For future studies, the buffy coat technique provides a more reliable means of screening individuals for trypanosome infections and clearly enhances the ability to detect low-level infections compared with standard whole blood smears. Similar techniques have been adopted and refined for detecting trypanosome infections in humans and livestock (Murray *et al*., 1977; Picozzi *et al*., 2002; Chappuis *et al*., 2005) from tropical regions where trypanosome infections can be severely debilitating and even fatal.

The prevalence of trypanosomes in our study (65%) is similar to what we previously reported from this population (56–62%; Davis and Hopkins, 2013; DuRant *et al*., 2015), but the refinement in our techniques used for detecting trypanosomes here suggests that the prevalence in earlier years may have been even higher than we initially reported. Our previous work used manual screening of 50 fields of view from whole blood smears (Davis and Hopkins, 2013; DuRant *et al*., 2015). It is possible that the prevalence of trypanosomes would have been higher in those previous years if we had concentrated them in the buffy coat. If leeches are in fact the vector for trypanosome transmission, a higher prevalence of trypanosomes in previous years would also be consistent with the higher prevalence of leeches in those same years. We also found that the prevalence of trypanosome infection increased in adults, a finding consistent with slow elimination of blood parasites and/or gradual accrual of infection over time (Gjini *et al*., 2010). Alternatively, adults could have higher contact rates with the vector of the trypanosomes (e.g. leeches) owing to ontogenetic differences in movement and/or microhabitat selection.

Our work here, in conjunction with our previous studies (Durant *et al*., 2015), suggests that leeches and trypanosomes provoke physiological responses by their hellbender hosts. We documented changes in TPPs and white blood cell profiles that were consistent with predictable responses to parasitic infections. Specifically, we found evidence that individuals harbouring leeches, and to a lesser degree trypanosome infections, had increased TPP compared with uninfected individuals after controlling for effects of body size. Inflammatory proteins (e.g. α, β and γ globulins), and consequently TPP, often increase in response to infection (Tatum *et al*., 2000; Cray *et al*., 2001; Zaias and Cray, 2002; Latimer *et al*., 2003). In mammals, increases in immunoglobulin and total protein have been documented in response to trypanosomiasis (Orhue *et al*., 2005). Likewise, in response to both types of parasites we found evidence that the percentage of neutrophils increased whereas the percentage of lymphocytes decreased, leading to greatly elevated N:L ratios in infected individuals (Table 2). In amphibians, neutrophils increase in the circulation in response to infection and inflammation (Wright and Whitaker, 2001; Claver and Quaglia, 2009). Concomitantly, the relative prevalence of amphibian lymphocytes decreases in blood in response to infection by being redistributed from the circulation into tissues such as skin and lymph nodes. Lymphoctyes are important for immunoglobulin production and for antibody-dependent, cell-mediated cytotoxicity (Hansen and Zapata, 1998; Wright and Whitaker, 2001; Campbell and Ellis, 2007; Allender and Fry, 2008). The percentage of circulating eosinophils also increased among individuals infected with either parasite. Eosinophils are important for innate immunity and are believed to be effective against some parasitic infections in amphibians (Mitchell, 1982).

The bactericidal capacity of plasma increased markedly (by 55%) among individuals harbouring leeches compared with individuals without leeches, which was contrary to our initial predictions and raises additional important questions. First, why does leech parasitism induce this response? It is possible that the leech bite itself is sufficient to elicit immunological responses integral to combating infection, such as increased production of complement proteins (Volanakis, 1995). However, because leeches are such efficient vectors of other parasites and pathogens (Sawyer, 1986; Mock, 1987; Barta and Desser, 1989; Raffel *et al*., 2006), it is also possible that up-regulation of plasma bactericidal capacity is a response to secondary pathogens and not to leeches *per se*. Last, because leech saliva contains many bioactive compounds (Salzet *et al*., 2000; Hildebrandt and Lemke, 2011), and it is believed that these compounds have systemic effects on hellbender physiology (DuRant *et al*., 2015), it is also possible that components of leech saliva modify innate immune responsiveness. Second, what is the physiological mechanism for increased killing capacity? In plasma, an array of antibodies, complement enzymes, lysozyme and acute phase proteins play important roles in bactericidal capacity, and some of these factors, (e.g. antibodies, complement and acute phase proteins) are inducible responses (Fearon and Locksley, 1996; Medzhitov and Janeway, 1997; reviewed by Matson *et al*., 2006). The fact that we found evidence that TPP increased in parasitized hellbenders is consistent with the possible induction of serological immune components.

Interestingly, the immune responses we evaluated *in vivo* and *in vitro* were more robust in adults than in juveniles. Although our sample size of infected juveniles was low, higher bactericidal capacity in adults than in juveniles mirrors a recent anecdotal result from our laboratory on hellbenders (Hopkins and DuRant, 2011). A number of studies on wildlife have detected increased innate immune responses in adults relative to juvenile age classes (e.g. Buehler *et al*., 2009; Wilcoxen *et al*., 2010; Palacios *et al*., 2011; Bull *et al*., 2012; Arriero *et al*., 2013), and in some cases, these responses continue to increase with body size/age in adults (Madsen *et al*., 2007; Sparkman and Palacios, 2009; Ujvari and Madsen, 2011; Palacios *et al*., 2013). Likewise, the redistribution of WBCs in response to infection can differ among vertebrate age classes (Shender *et al*., 2002; Pavlak *et al*., 2005). A number of possible mechanisms have been proposed to explain age-specific changes in immune function. For example, ontogenetic changes in the expression of genes associated with immunity may lead to increased immune responses and differences in disease resistance between age classes (Bull *et al*., 2012). Additionally, slow development of immune function may reflect life-history trade-offs, where young animals invest more energy and/or nutrients into somatic growth than towards immunity to offset other sources of juvenile mortality (e.g. size-dependent predation) and to decrease the time required to reach sexual maturity (Hasselquist and Nilsson, 2012; Arriero *et al*., 2013). Regardless of the mechanisms underlying age-dependent immunity in hellbenders, reduced immune responsiveness in young hellbender age classes could be important in understanding how population demography relates to differential ontogenetic responses to pathogens. This is especially important considering that young age classes are notably absent from streams where hellbender population declines have occurred (Wheeler *et al*., 2003; Foster *et al*., 2009; Burgmeier *et al*., 2011), and disease has been suggested as contributing to declines in some of these populations (Briggler *et al*., 2007a; USFWS, 2011a). Clearly, to determine whether early immune function can influence juvenile recruitment, studies on other aspects of immunity and responses to pathogens across stages of ontogeny are ultimately needed.

An important methodological outcome of our study was the revelation that plasma maintained remarkably stable bactericidal capacity after being stored for 2 months at −80°C. Previous work has shown that bactericidal capacity of bird plasma and whole blood declines within weeks when stored at −20°C (Liebl and Martin, 2009) and within several months when stored at −80°C (DuRant, 2011). However, little is known about the intermediate temporal stability of plasma bactericidal capacity when plasma is stored at ultracold temperatures, let alone the stability of plasma from ectothermic vertebrates. As a result, many researchers have recently relied on freshly collected plasma to avoid confounding effects of freezer storage. Thus, our findings have significant practical implications for future work on hellbenders because assays can be run on plasma frozen for at least 2 months. However, it remains unknown whether our findings are applicable to other situations. It is possible that the temporal stability of plasma varies among vertebrate species and among pathogens being tested in the assay, and thus future investigators should validate these conditions for the species and pathogen of interest.

Although we found evidence that immune parameters were influenced by infection, there was no evidence to support our hypothesis that trypanosomes might cause anaemia in hellbenders. Red blood cell count, PCV and haemoglobin concentrations were all positively correlated with one another and loaded heavily on PC1, but showed no relationship with trypanosome infection. However, we found that body size had a positive influence on these RBC parameters. Previous studies in other vertebrates have demonstrated similar positive relationships between body size/age and RBC parameters related to oxygen-carrying capacity and suggest that fundamental differences in juvenile and adult physiology contribute to these allometric relationships (Pough, 1978, 1980; Hou and Burggren, 1989; Dunlap, 2006; but see Garland, 1984).

Our study provides novel insights into the physiological responses of hellbenders exposed to endo- and ectoparasites, as well as co-infection with both, and raises important questions about how these parasites might influence the health of one of the most intriguing and imperilled amphibians in North America. Given that this host–parasite system was only described recently (Davis and Hopkins, 2013; Hopkins *et al*., 2014), many fundamental questions remain unanswered. For example, it currently remains unclear whether *P. appalachiensis* is a hellbender specialist and whether it is in fact the primary vector for trypanosome infections to hellbenders. Elucidating these relationships is central to understanding vector-borne diseases and whether trypanosome transmission occurs among different host species. It is also unclear whether leeches transmit other pathogens, such as viruses, fungi and bacteria, to hellbenders as they do in other systems (Sawyer, 1986; Mock, 1987; Barta and Desser, 1989; Raffel *et al*., 2006). This is particularly important given that leeches and trypanosomes are so prevalent in Ozark hellbenders, where disease is believed possibly to contribute to their declines (Briggler *et al*., 2007a, b, 2008; Bodinof *et al*., 2012). Finally, all of the physiological responses to infection documented here are consistent with adaptive immune responses, but our recent work suggests maladaptive suppressive effects of leeches on normal adrenocortical responses (DuRant *et al*., 2015). Thus, additional work needs to be done to address how these parasites influence other physiological processes, especially given the effects of leeches on their hosts' physiology and the array of multisystemic effects that some trypanosomes can have, particularly on the cardiovascular, haemolymphatic, immune, musculoskeletal and nervous systems (Meurs *et al*., 1998; Berriman *et al*., 2005; Blum *et al*., 2006). Ultimately, determining whether infections influence growth, reproduction and/or survival will be critical to hellbender conservation.

## Supplementary material

Supplementary material is available at *Conservation Physiology* online.

## Funding

This work was supported by the Virginia Department of Game and Inland Fisheries and the Fralin Life Science Institute at Virginia Tech.

## Supplementary Material

Supplementary Data
