# Supplementary material for: Haematological and immunological characteristics of eastern hellbenders (Cryptobranchus alleganiensis alleganiensis) infected and co-infected with endo- and ectoparasites
Source: Conserv Physiol. 2016 Mar 21;4(1):cow002. doi: 10.1093/conphys/cow002 (PMC4801058; doi:10.1093/conphys/cow002)
Supplement: Supplementary Data [file cow002supp.zip › cow002supp_fig1.docx]

**Supplemental Figure 1.** Influence of body size (snout-vent length [SVL]) on blood parameters of eastern hellbenders (*Cryptobranchus alleganiensis*). *Panel A* shows the positive relationship between SVL and red blood cell parameters positively loading on Principal Component 1 (packed cell volume, red blood cell count, and hemoglobin concentration). *Panel B* shows the relationship between SVL and total plasma protein (g/ 100 ml; estimated total solids).
