# Supplementary material for: Haematological and immunological characteristics of eastern hellbenders (Cryptobranchus alleganiensis alleganiensis) infected and co-infected with endo- and ectoparasites
Source: Conserv Physiol. 2016 Mar 21;4(1):cow002. doi: 10.1093/conphys/cow002 (PMC4801058; doi:10.1093/conphys/cow002)
Supplement: Supplementary Data [file cow002supp.zip › cow002supp_table1.docx]

**Supplemental Table 1.** Factor loading matrix for principle components describing red blood cell and white blood cell parameters in eastern hellbenders.

| **RBC parameter** | **RBCPC1** | **RBCPC2** |  | **WBC parameter** | **WBCPC1** |
| --- | --- | --- | --- | --- | --- |
| Hb average | 0.866 | -0.053 |  | % neutrophil | 0.821 |
| PCV | 0.916 | 0.394 |  | % lymphocyte | -0.981 |
| RBC/100ml | 0.882 | -0.331 |  | % eosinophil | 0.643 |
| MCV | -0.231 | 0.855 |  | N:L ratio | 0.967 |
| MCHC | -0.143 | -0.748 |  |  |  |
|  |  |  |  |  |  |
| *Eigen value* | 2.44 | 1.56 |  | *Eigen value* | 2.983 |
| *% variance* | 48.79 | 31.16 |  | *% variance* | 74.59 |
